# Fibro-Cystic Tumors of the Jaw

**Published:** 1885-03

**Authors:** W. F. Westmoreland

**Affiliations:** Professor of Surgery in the Atlanta Medical College, Atlanta, Georgia


### FIBRO-CYSTIC TUMORS OF THE JAW.

A CLINICAL LECTURE.

By W. F. WESTMORELAND, M. D.,
Professor of Surgery in the Atlanta Medical College, Atlanta, Georgia.

Reported for the Atlanta Medical and Surgical Journal.

This case, gentlemen, presents, as you see, a rather hideous appearance. The man, a large, healthy looking fellow, twenty-eight years old, has a large tumor, as you see, of the lower jaw, involving the symphysis and extending back on either side to a point about midway between the symphysis and the angle. He says the growth began when he was fifteen years old. If that is true, it has been growing for thirteen years.

The tumor now projects forward and downward fully three inches. It gives him no pain. He seeks to have it removed because of the inconvenience it causes him.

It is not usual, gentlemen, that this tumor should have existed so long. Not infrequently they begin in very early life, though they sometimes begin late, as I have seen in more than one case where there was no appearance of the tumor until after forty years. I desire that you notice especially the size and appearance of the tumor and the sensation imparted to the fingers when hard pressure is made on it. This sensation I cannot better describe to you than to say that it resembles the peculiar crackling noise made by parchment whenever firm pressure is made, as you see me now making. This is a characteristic symptom of these tumors, and is caused by the giving way of the thin shell of bone which forms the out wall of the tumor.

What is this tumor? Some authors say it is dentigerous, that it is produced by an undeveloped or impacted tooth; that a cyst is formd by this irritation, and from it springs this tumor. I take a different view of it, however, and say that it is a

FIBRO-CYSTIC TUMOR.

I take this position from the teaching received from the great Nelaton. He claimed that the teeth had nothing to do with the formation of such tumors, and I fully agree with him, and believe that I can convince any one of the correctness of the views as held by him.

One thing I am certain of, that the tumor is not malignant, and would never become so if allowed to remain. It is true that it will continue to grow if left alone, just as it has done in the past, but it is not malignant and never would become so.

These tumors may form on any part of either jaw, and if allowed to remain, grow sometimes to enormous size. They are readily cured when small if an opening is made into them and they are well drained and suppuration is set up. But when they have reached the size of this one nothing is left for us to do but to remove the entire bone involved.

It is always important in operations on the jaw that as much of the cutting as can be, should be done on the inside of the mouth. This, as you know, is to prevent the unsightly scars that are left by incisions through the skin. In this case we will attempt the operation by doing all the cutting on the inside. You notice that I begin the dissection at the symphysis, and with the bistoury I cut through the mucous membrane on either side to a point near the angle of the jaw. I now take Sayre's oyster knife, which, as you see, has no cutting edge, and I tear away the tissues, so to speak, keeping the instrument well against the periosteum. When I strike a muscle that does not readily yield to the oyster knife I divide it with the bistoury, and again take the oyster knife, until I have dissected the tissues away sufficiently to allow the use of the chain saw. This saw must be introduced under the bone by means of a needle, and after trial you perceive the needle is not long enough. Now we try an ordinary silver probe with an "eye" to it, but find it will not answer. It will never do to postpone the operation until we can send to the city for another needle, and we must resort to some other means of dividing the bone. Hence we use the ordinary bone forceps. We begin the cut from the alveola border, about midway between the symphysis and the angle, and cut through to the canal, then with chisel and mallet we split the tumor off, leaving the lower border of the bone. Now we can, without difficulty, pass the chain saw beneath the bone and sever it on either side a little anterior to the cuts made by the forceps from the alveola border. Now the entire mass has been removed, and you see, with the lip drawn back to its proper place, the patient presents a very good appearance. You will notice there is considerable hemorrhage, but this, we think, we can control without the application of any ligatures. The hemorrhage has now ceased, as you see, and now the dressing, which will consist of the introduction of a small amount of absorbent cotton into the place occupied by the tumor, and a strip of adhesive plaster placed under the chin and carried up the sides of the face to support the parts, and our operation is finished without a scratch on the face or the ligation of a single vessel.

This is the thirty-fourth case of this kind, gentlemen, upon which I have operated, and I have never known one to return or fail to recover perfectly.

There is one thing, gentlemen, that you should look to well before beginning any operation, and that is to see and know for yourselves, before you begin, that you have every instrument ready, so there may be no unnecessary delay. This I must confess I did not do in this case, or we would not have had the difficulty of dividing the bones we have had. If the chain saw could have been used we would have finished the operation in one-fourth the time it required by the slow and bunglesome process of forceps and chisel.

